# Choroidal vascularity index in different types of central serous chorioretinopathy: A meta-analysis

**DOI:** 10.1371/journal.pone.0289186

**Published:** 2023-07-27

**Authors:** Yihao Xia, Xiaodong Li, Jiaqi Zhang, Xuejun Xie

**Affiliations:** 1 Eye School of Chengdu University of Traditional Chinese Medicine, Chengdu, Sichuan, China; 2 The First Affiliated Hospital of Guizhou University of Traditional Chinese Medicine, Guiyang, Guizhou, China; 3 Department of Ophthalmology, Hospital of Chengdu University of Traditional Chinese Medicine, Chengdu, Sichuan, China; Kobe University Graduate School of Medicine School of Medicine: Kobe Daigaku Daigakuin Igakukei Kenkyuka Igakubu, JAPAN

## Abstract

**Purpose:**

To evaluate the choroidal vascularity index (CVI) in different types of central serous chorioretinopathy (CSC), healthy control eyes, and fellow eyes.

**Methods:**

Relevant studies published up to January 2023 were identified by searching multiple databases, including PubMed, Embase, Web of Science, Cochrane Library, and China National Knowledge Infrastructure (CNKI). Studies investigating the difference in CVI between CSC and control eyes were included. Data from these studies were analyzed using Stata (version 17) software. Weighted mean difference (WMD) and 95% confidence interval (95%CI) were calculated for the CVI in CSC eyes, control eyes, and fellow eyes.

**Results:**

The meta-analysis included 15 studies, with 213 acute CSC eyes, 153 chronic CSC eyes, 92 uncategorized CSC eyes, 40 resolved CSC eyes, 409 eyes of normal healthy controls, and 318 fellow eyes. The result revealed that CVI was higher in acute CSC eyes (WMD = 5.40, 95%CI = 2.36–8.44, P = 0.001) compared to control eyes. Also, CVI in chronic CSC eyes was higher than in control eyes (WMD = 1.26, 95%CI = 0.03–2.49, p = 0.046). The fellow eyes of acute CSC had a higher CVI when compared to control eyes (WMD = 2.53, 95%CI = 0.78–4.28, p = 0.005). There was no significant difference in CVI between acute and chronic CSC eyes (WMD = 0.75, 95%CI = -0.31–1.82, P = 0.167). In the sub-analysis based on the area selected for CVI calculation, the WMDs in the whole image subgroups were lower than the main analysis for the comparisons of fellow eyes of acute CSC and control eyes, acute CSC eyes and control eyes, and acute CSC eyes and fellow eyes. In the macular area subgroups, the WMDs were higher than in the whole image subgroups, suggesting a potential regional variation of CVI in CSC eyes.

**Conclusions:**

The results demonstrated that CVI is increased in CSC eyes and fellow eyes of acute CSC. There is no significant difference in CVI between acute and chronic CSC eyes. The area selected for CVI calculation can influence the outcome, which requires further clinical research to clarify.

## Introduction

Central serous chorioretinopathy (CSC) is a self-limiting retinal disorder characterized by the accumulation of subretinal fluids between the neurosensory retinal layer and the retinal pigment epithelium (RPE), resulting in serous retinal detachment(SRD) [1]. Although CSC is commonly unilateral, there have been reports of bilateral cases [2, 3]. As part of the pachychoroid disease spectrum, contralateral manifestations such as choroidal hyperpermeability, dilated choroidal vessels, and a thickened choroid have also been observed in CSC patients [4, 5]. These changes suggest that both eyes of CSC patients may undergo similar pathological changes. According to studies published in recent years, the disease is more prevalent in middle-aged males than females [6, 7]. Identified risk factors for CSC include steroid use, pregnancy, smoking, obstructive sleep apnea, H. pylori infection, and Type A personality [8, 9].

CSC was initially considered a type of retinitis by von Graefe [10] in 1866. It is now thought that the subretinal fluid in CSC patients leaks from the RPE membrane, associated with hyperpermeable choroidal dysfunction. This theory is supported by findings derived from the development of fundus fluorescein angiography (FFA), indocyanine green angiography (ICGA), and optical coherence tomography (OCT). Gass [11] proposed that hyperpermeability of the choroidal vessels and increased hydrostatic pressure may induce dysfunction of the RPE, as observed in FFA images. The findings of ICGA further supported this theory by revealing abnormal hyperfluorescence indicating choroidal vascular hyperpermeability, as well as congestion and anastomosis of the vortex vein, suggesting the outflow of the choroid may be restrained [12–14]. OCT has also revealed dilated vessels and thickening of the choroid in CSC patients [15, 16]. The advent of advanced OCT technology, including enhanced depth imaging (EDI)-OCT and swept source (SS)-OCT, has provided a better way to assess the structure and morphological changes of the choroid and prompted the development of new parameters [17], such as the choroidal vascularity index (CVI).

In 2014, Sonoda [18] and colleagues introduced the concept of the ratio of luminal to choroidal area. They differentiated the luminal area (LA) of choroidal images obtained by EDI-OCT using the Niblack autolocal thresholding binarization method in ImageJ software. In contrast to Sonoda’s method, which selects the choroidal area before binarization, in 2016, Agrawal [19] modified this method by proceeding with binarization before selecting the choroidal area, and they calculated the ratio of the LA to the total choroidal area and called it CVI. In recent years, more studies have used CVI as an indicator to evaluate the choroid in healthy or diseased eyes [20, 21].

CVI is thought to be less affected by physiological changes than other imaging parameters, such as choroidal thickness, and is thus considered a stable imaging biomarker in some studies [22, 23]. As a result, CVI has become a promising tool for studying the pathological mechanism of CSC. However, despite some studies reporting a higher CVI ratio in CSC eyes and fellow eyes, inconsistent results have been observed, with some studies revealing similar outcomes between CSC eyes or fellow eyes and healthy normal eyes [24, 25]. These discrepancies may be attributed to differences in patient characteristics and imaging analysis protocols used in different studies. Although some patients can recover from the disease spontaneously, there is still a portion of patients who develop chronic CSC. Differences between acute and chronic CSC eyes in CVI need further research. With the available data, our meta-analysis aims to provide a more comprehensive understanding of CVI in different types of CSC patients.

## Materials and methods

The meta-analysis followed the recommendations of the Preferred Reporting Items for Systematic Reviews and Meta-Analyses (PRISMA) checklist (S1 Table) [26]. This study was registered. Protocol and registration information is available on Prospero (Registration number: CRD42022379484).

### Search strategy

A comprehensive search of the PubMed, EMBASE, Cochrane Library, Web of Science, and China national knowledge infrastructure (CNKI) databases was conducted by two independent reviewers using items ’central serous chorioretinopathy’, ’choroidal vascularity’ and ’choroidal vascularity index’. The titles, keywords, and abstracts of the resulting studies were screened to identify potentially relevant ones. The full text was then reviewed for each relevant study, and those that met the inclusion criteria were selected. The final search was conducted on January 15, 2023. Only studies written in English or Chinese were included. Any disagreements about the inclusion of studies were resolved by discussion.

### Inclusion and exclusion criteria

Studies were considered eligible for inclusion in this meta-analysis if they met the following criteria: (1) Studies comparatively evaluated the CVI in CSC patients and normal control eyes or fellow eyes. (2) Observational studies, including retrospective, prospective, and cross-sectional studies. (3) CSC patients who were diagnosed using recognized criteria. (4) Available raw data with mean value and standard deviation (SD). Exclusion criteria: (1) Animal studies, conference articles, and reviews. (2) Duplicated articles. (3) Studies that included treated patients with a history of laser or ocular surgery.

### Data extraction

The selection of studies for this meta-analysis was conducted in three steps, with two independent reviewers involved in the process. The first step involved screening the titles, abstracts, and keywords of relevant studies. Then the full text of the literature was screened to determine if it met the inclusion criteria. Finally, disagreements about the included studies were resolved through discussion between the two independent reviewers.

Data extraction was carried out by two independent reviewers who extracted data independently. The two reviewers then reviewed the extracted data together to ensure accuracy and completeness. The following characteristics were extracted: study design, authors, publication year, country, instrument, number of eyes (total/acute/chronic/control/fellow eyes), mean age, selected study area, CVI calculation protocol, binarization method, and main outcome of CVI. The data was recorded using spreadsheet software (WPS software), and the extraction was done manually.

### Qualitative assessment

Two independent reviewers assessed the quality of the included studies. For cohort or case-control studies, the Newcastle-Ottawa Scale (NOS) was used [27], which comprises eight items in three parts: selection bias, comparability bias, and outcome/exposure bias. A score above four points was required for studies to be included in the meta-analysis. For cross-sectional studies, the Joanna Briggs Institute (JBI) Critical Appraisal Checklist for analytical cross-sectional study assessment tool was used [28]. The scale includes 8 questions and up to 8 points, and only studies with a score above 4 points were included in the meta-analysis. Disagreements between reviewers were resolved through discussion.

### Statistical analysis

The statistical analysis was performed using Stata software (Version 17, Stata Corp, College Station, Texas). Weighted mean difference (WMD) was used to assess mean values and standard deviations (SDs) as continuous variables, with a 95% confidence interval (95%CI). Statistical significance was determined when P<0.05. I^2 was calculated to assess the heterogeneity between studies and subgroups. When I^2>50%, the random effects model was used for the data analysis; otherwise, the fixed effects model was used. The results of the analysis were visually presented in forest plots. In the presence of high heterogeneity, sensitivity analysis was conducted using the leave-one-out method in Stata. Sub-analysis was conducted based on the type of CSC and the study areas selected in the included studies. Egger’s test was used to identify potential publication bias through Stata software. Bias was identified when p<0.05.

## Result

### Overall characteristics of selected studies

**Fig 1:**
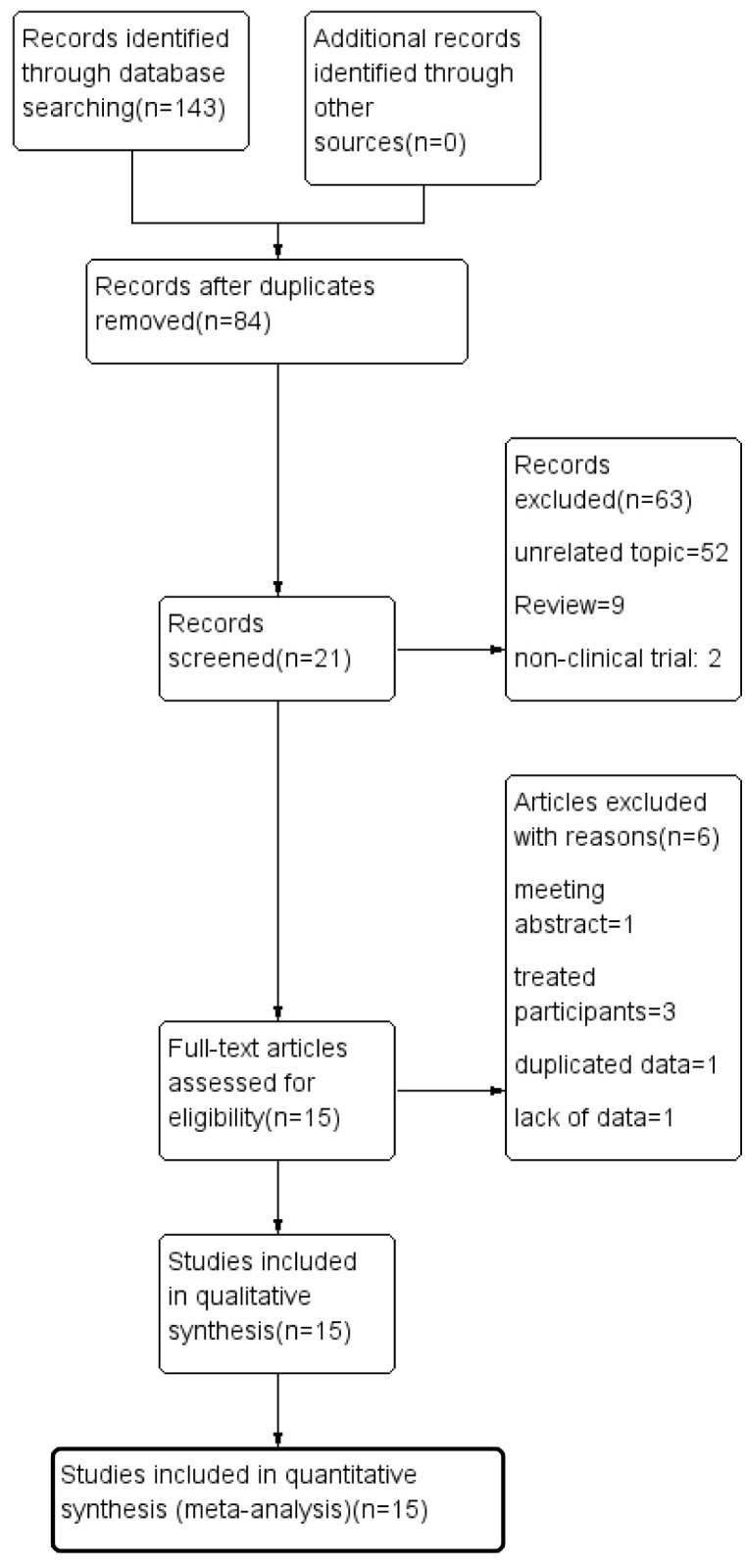
Flog diagram of the meta-analysis. Flow diagram about selection of included studies.

After removing duplicated articles, a total of 84 studies were initially identified from the databases Pubmed, Embase, Web of Science, Cochrane Library, and CNKI. Through screening the title, keywords, abstract, and full text, 69 studies were removed according to the inclusion and exclusion criteria. Thus, 15 studies were included in the meta-analysis [23–25, 29–40]. 3 studies about the comparison between acute CSC and chronic CSC, 7 studies about the comparison between acute CSC and control eyes, 3 studies about the comparison between chronic CSC and control eyes, and 5 studies about the comparison between uncategorized CSC and control eyes. A flow diagram of the search and selection procedure is shown below (Fig 1). The analysis included 213 acute CSC eyes, 153 chronic CSC eyes, 92 uncategorized CSC eyes, 40 resolved CSC eyes, 409 eyes of normal healthy controls, and 318 fellow eyes. Six studies used Heidelberg SD-OCT (Heidelberg Engineering, Heidelberg, Germany) in EDI mode [24, 29, 30, 32, 37, 38]. One study used SS-OCTA PLEX Elite 9000 (Carl Zeiss Meditec Inc, Dublin, CA) [31]. One study used EDI-OCT RTVue XR 100 (Optovue, Inc., Fremont, CA, USA) [33]. One study employed EDI-OCT without specifying the exact instrument information [23]. Four studies used SS-OCT Topcon DTI Trition (Topcon Corporation, Tokyo, Japan) [25, 34–36]. One study used OCTA BM400K (TowardPi Medical Technology Co., Ltd., Beijing, China) [40]. One study used SS-OCT VG200 (SVision Imaging, Ltd., Luoyang, China) [39]. The quality assessment of the included studies was performed using the NOS for case-control and cohort studies and the JBI for cross-sectional studies. Among the case-control and cohort studies, seven had moderate quality scores ranging from 4 to 6, while four had high quality scores of 7. One cross-sectional study received a high quality score of 8, while the remaining three studies had moderate quality scores ranging from 5 to 6. The characteristics of all included studies are summarized in Table 1.

**Table 1:** Characteristics of included studies.

Study/year	Design	Country	OCT instrument/manufacturer	Study area	CVI calculation method	Binarization method	Number of eyes*	Mean age^#^	NOS/JBI Scores
Agrawal/ 2016	Retrospective	India	EDI-OCT/ Heidelberg Spectralis	Macular 1500㎛	Modified Sonoda`s method(FIJI)	Niblack autolocal threshold(FIJI)	32/32/-/30/27	42.2±5.85/-/-/37.4±6.5	7
Aslam/2022	Retrospective	UK	EDI-OCT/ Heidelberg Spectralis	Macular 3000㎛	Aslam`s automated method(Matlab)	-	40/-/-/32/-	48±11.1/-/-/50.5±14.1	6
Borrelli/ 2022	Cross- sectional	Italy	SS-OCTA/ PLEX Elite Carl zeiss	Macular 1500㎛	Sonoda`s method(FIJI)	Niblack autolocal threshold(FIJI)	-/-/-/30/60	47.5 ± 9.9/-/-/50.7 ± 10.8	6
Demirel/2020	Cross-sectional	Turkey	EDI-OCT/ Heidelberg Spectralis	Macular 3000㎛	Sonoda`s method(ImageJ)	Niblack autolocal threshold(ImageJ)	-/- /- /26/36	-	6
Faghihi/ 2021	Retrospective	Iran	EDI-OCT/ RTVue XR 100 Optovue	Whole image	Sonoda`s method(FIJI)	Niblack autolocal threshold(FIJI)	44/-/44/40/40	50.6 ± 11.2/-/50.6 ± 11.2/50.2 ± 9	6
GOUD/2021	Prospective	India	SS-OCT/ Topcon DTI Trition	Whole image	Agrawal`s automated method	-	29/14/15/13/14	-/34.92±7.32/42.33±5.08/34.46±10.12	7
Hwang/2022	Retrospective	Korea	SS-OCT/ Topcon DTI Trition	Whole image	Modified Sonoda`s method(ImageJ)	Niblack autolocal threshold(ImageJ)	20/20/-/20/20	-	6
Kim YH/2021	Retrospective	Korea	SS-OCT/ Topcon DTI Trition	Macular 1500㎛	Sonoda`s method(ImageJ)	Niblack autolocal threshold(ImageJ)	103/31/32/50/-	53.7 ± 11.4/48.3 ± 10.1/58.4 ± 8.3/55.9±11.4	6
Kim RY/ 2020	Retrospective	Korea	SS-OCT/ Topcon DTI Trition	Macular 1500㎛	Modified Sonoda`s method(ImageJ)	Niblack autolocal threshold(ImageJ)	78/29/49/-/-	-/42.0±7.0/46.7 ± 7.4/-/-	5
Sahoo/2021	Retrospective	Italy/USA	EDI-OCT/ Heidelberg Spectralis	Three-dimensional En face	Goud`s automated method	-	20/7/13/20/20	48±11.1/-/-/-	4
Scarinci/2021	Cross-sectional	Italy	EDI-OCT/ Heidelberg Spectralis	Whole image	Sonoda`s method(ImageJ)	Niblack autolocal threshold(ImageJ)	17/17/-/17/-	48±7/48±7/-/50±11	8
Tatti/2022	Retrospective	Italy	EDI-OCT/ Heidelberg Spectralis	En face	Singh`s method	particle swarm optimization thresholding	20/-/-/20/20	50.7 ± 9.96/-/-/48.8 ± 3.5	6
Wang/2019	Retrospective	China	EDI-OCT/-	Macular 1500㎛	Modified Sonoda`s method(ImageJ)	Niblack autolocal threshold(ImageJ)	31/31/-/31/31	41.90±7.96/41.90±7.96/-/42.06±8.71	7
Yang/2020	Cross-sectional	China	SS-OCT/VG200	Three- dimensional En face	-	-	32/32/-/48/18	48.8±11.9/48.8±11.9/-/50.7±9.3	5
ZENG/2022	Proespective	China	SS-OCTA/ BM400K TowardPi	Three- dimensional En face	Built-in software	-	32/-/-/32/32	45.03 ± 11.394/-/-/46.79 ± 8.510	7

*Central serous chorioretinopathy eyes/acute eyes/chronic eyes/control eyes/fellow eyes

#Central serous chorioretinopathy eyes/acute eyes/chronic eyes/control eyes

### Meta-analysis result

#### CVI in CSC eyes and control eyes

**Fig 2:**
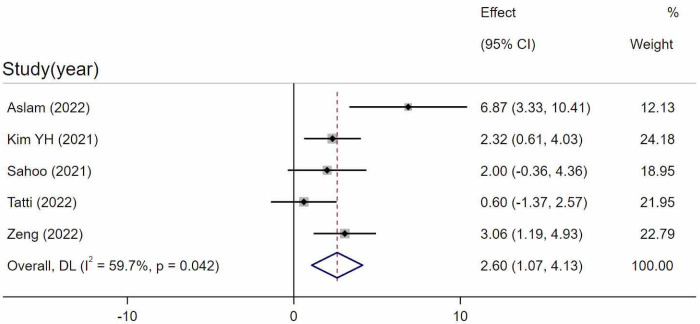
Comparison between uncategorized CSC and control eyes. Random-effects model evaluating the differences between uncategorized CSC and control eyes. SD, standard deviation; IV, inverse variance; CI, confidence interval.

Five studies were included in calculating CVI between uncategorized CSC and control groups. Three studies [24, 30, 40] did not state the exact number of acute or chronic eyes. Collectively they included 92 eyes of CSC and 84 eyes of controls. Two studies [36, 37] included 38 eyes of acute CSC, 45 eyes of chronic CSC, 40 eyes of resolved CSC, and 70 eyes of controls and calculated the overall outcome of all included CSC eyes. The comparison showed that CVI in uncategorized CSC eyes was statistically significantly higher than the control eyes (WMD = 2.60, 95%CI = 1.07–4.13, P = 0.001, I^2 = 59.7%) (Fig 2). A sensitivity analysis was carried out, but the significance of the outcome was stable. Egger’s test revealed that no significant publication bias existed (p = 0.231).

**Fig 3:**
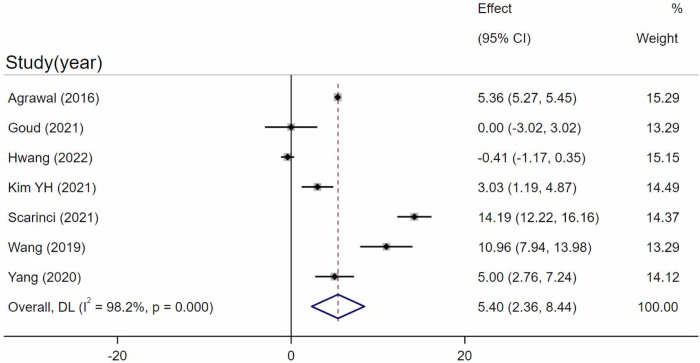
Comparison between acute CSC and control eyes. Random-effects model evaluating the differences between acute CSC and control eyes.SD, standard deviation; IV, inverse variance; CI, confidence interval.

Seven studies [23, 25, 29, 34, 36, 38, 39] compared CVI in acute CSC eyes and control eyes. After combining the outcomes of the included studies, the CVI in acute CSC was statistically significantly higher than in the control eyes (WMD = 5.40, 95%CI = 2.36–8.44, P = 0.001, I^2 = 98.2%) (Fig 3). Despite the high heterogeneity (I^2 = 98.2%), sensitivity analysis of leave-one-out did not substantially change the statistical significance. Egger’s test revealed that no significant publication bias existed (p = 0.725).

**Fig 4:**
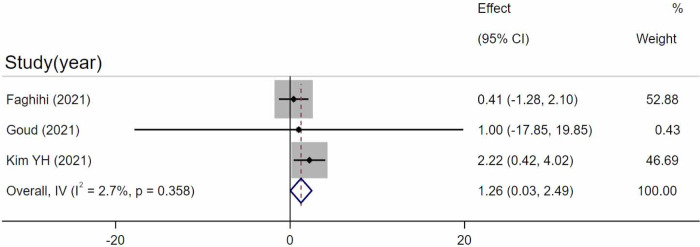
Comparison between chronic and control eyes. Random-effects model evaluating the differences between chronic and control eyes. SD, standard deviation; IV, inverse variance; CI, confidence interval.

Three studies [33, 34, 36] compared CVI in chronic CSC eyes and control eyes. As a result, chronic CSC eyes had a significantly higher CVI ratio compared to control eyes (WMD = 1.26, 95%CI = 0.03–2.49, p = 0.046, I^2 = 2.7%) (Fig 4). Egger’s test revealed that no significant publication bias existed (p = 0.981).

#### CVI between acute CSC and chronic CSC eyes

**Fig 5:**
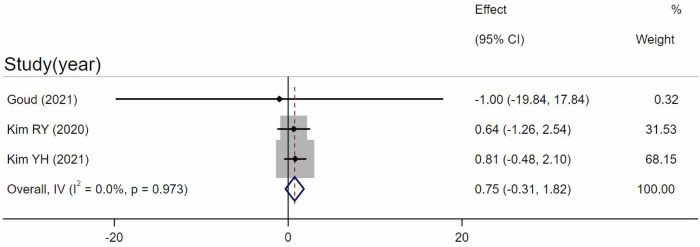
Comparison between acute and chronic eyes. Meta-analysis evaluating the differences between acute and chronic eyes. SD, standard deviation; IV, inverse variance; CI, confidence interval.

Three studies [34–36] compared the CVI between acute and chronic CSC eyes. After combining the data, the CVI in acute CSC was slightly higher than that in chronic eyes. However, the difference was not statistically significant (WMD = 0.75, 95%CI = -0.31–1.82, P = 0.167, I^2 = 0%) (Fig 5). Egger’s test revealed that no significant publication bias existed (p = 0.250).

#### CVI in CSC eyes and fellow eyes

**Fig 6:**
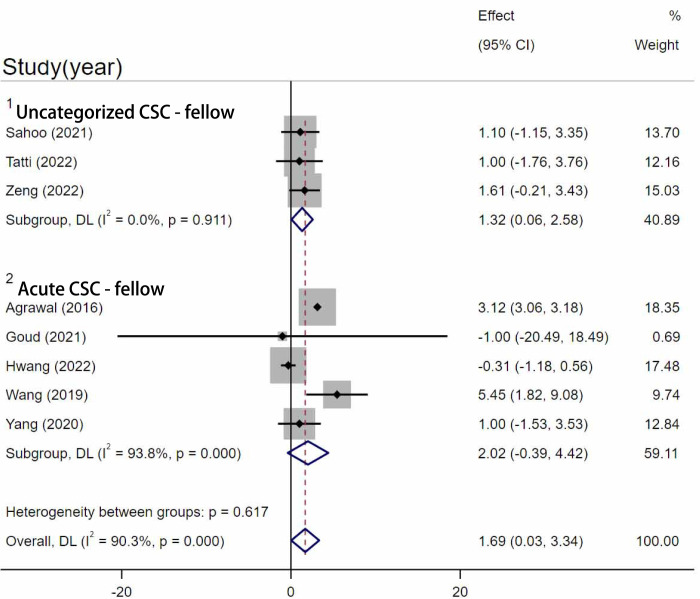
Comparison between CSC and fellow eyes. Meta-analysis evaluating the differences between CSC and fellow eyes. SD, standard deviation; IV, inverse variance; CI, confidence interval.

Nine Studies [23–25, 29, 33, 34, 37, 39, 40] compared the difference in CVI between CSC eyes and fellow eyes. Due to high heterogeneity, a subgroup analysis was conducted. The twelve studies were categorized into three subgroups based on the type of CSC they included (Fig 6). In the comparison of uncategorized CSC subgroup, the result showed a statistically significant difference that uncategorized CSC eyes had a higher CVI than the fellow eyes (WMD = 1.32, 95%CI = 0.06–2.58, I^2 = 0%, P = 0.039) (Fig 6). In the acute subgroup, CVI in acute CSC eyes was higher than in fellow eyes, but the difference was not statistically significant (WMD = 2.02, 95%CI = -0.39–4.42, I^2 = 93.8%, P = 0.1). Only one study conducted by Faghihi [33] studied the CVI between the chronic CSC and their fellow eyes, and their study also revealed no significant difference between the two groups. Egger’s test revealed that no significant publication bias existed (p = 0.133).

#### CVI in fellow eyes and control eyes

**Fig 7:**
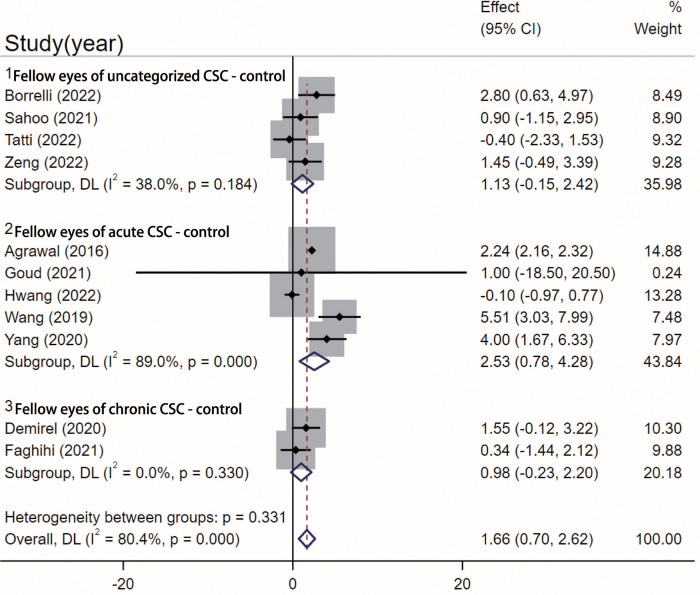
Comparison between fellow eyes and control eyes. Meta-analysis evaluating the differences between fellow eyes and control eyes. SD, standard deviation; IV, inverse variance; CI, confidence interval.

Eleven studies [23–25, 29, 31–34, 37, 39, 40] compared CVI between fellow eyes and control eyes. Subgroup analysis was performed based on the type of CSC included. In the uncategorized CSC subgroup, no statistically significant difference was found between fellow eyes and control eyes (WMD = 1.13, 95%CI = -0.15–2.42, I^2 = 38%, p = 0.083) (Fig 7). In the fellow eye of the acute CSC subgroup, a statistically significant difference was observed, with higher CVI in fellow eyes compared to control eyes (WMD = 2.53, 95%CI = 0.78–4.28, I^2 = 89%, p = 0.005). In the fellow eye of the chronic CSC subgroup, no statistically significant difference was found compared to control eyes (WMD = 0.98, 95%CI = -0.23–2.20, I^2 = 0%, p = 0.113). Egger’s test revealed no significant publication bias in these twelve studies (p = 0.286).

#### Sub-analysis based on different study regions

**Fig 8:**
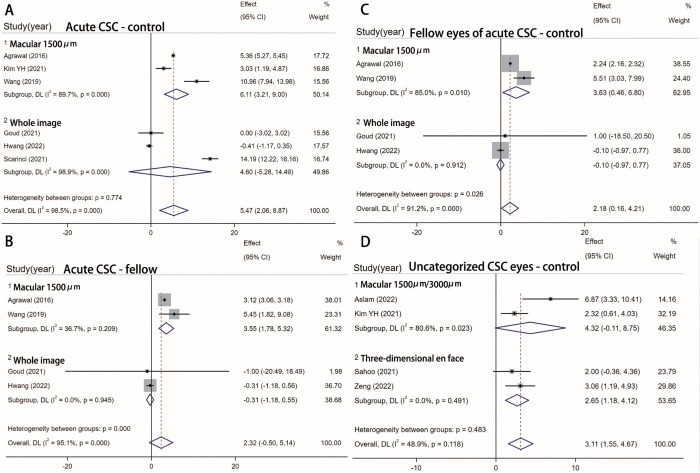
Sub-analysis based on the study area. Sub-analysis based on the study area. SD, standard deviation; IV, inverse variance; CI, confidence interval.

CVI has been widely used in various diseases, but selecting a specific choroid region is required, and different regions may yield different results. A sub-analysis was conducted based on the different study areas to address this issue. However, due to the limited number of studies included, only three subgroups were set: the macular subgroup and whole image subgroup, which calculated CVI in B-scan image, and the three-dimensional en face subgroup. In macular subgroups, the significance changed in a few subgroups (Fig 8B, 8D). In subgroups of the whole image, WMD suggested a lower result compared to main analysis (Fig 8A-8C), and all comparisons revealed no statistically significant differences. Moreover, the WMD in macular subgroups was higher than in whole image subgroups in all comparisons. The result indicated that restricting the calculation area to the macula resulted in a higher CVI ratio, which may be associated with the predominant involvement of the macula in CSC. The comparison between the three-dimensional en-face subgroup and the macular subgroup also showed that the WMD was higher in the macular subgroup (Fig 8D).

## Discussion

In this meta-analysis, we reviewed 15 studies to compare the CVI among different types of CSC eyes and evaluate the differences between fellow eyes and control eyes. Our results revealed that CSC eyes exhibited a significantly higher CVI ratio than normal control eyes. And uncategorized CSC eyes had a higher CVI compared to fellow eyes. The fellow eyes of patients with acute CSC also displayed a higher CVI ratio when compared to control eyes. However, we found no statistically significant difference when comparing acute and chronic CSC eyes.

CVI has been widely utilized in studies of various fundus diseases. Several studies have indicated that CVI is a reliable indicator for investigating pachychoroid diseases like CSC [23, 41]. In recent years, the number of clinical studies on CVI in CSC patients has increased. Some studies have revealed significant differences in CVI between CSC and control groups, while others have not. These inconsistencies were also observed in some parts of our meta-analysis, which may be attributed to variations in the selected study area. Some studies reported no significant differences in CVI between different choroidal areas. Agrawal [41] found a similar CVI ratio in the subfoveal region and other regions in B-scan images. Kim YH [36] also found no differences in CVI calculated on volumetric ETDRS scales. However, our sub-analysis outcome suggested that the region selected for CVI calculation may affect the final results. In a wider study area under B-scan images, the difference in CVI between CSC groups and control or fellow eyes tended to be smaller. A potential explanation for this finding is that CSC primarily affects the macula. Including non-lesion areas around the macula in the study may reduce the differences in CVI between CSC eyes and normal or fellow eyes, resulting in a lower result of WMD. However, due to the limited number of included studies and potential methodological heterogeneity, caution is needed when concluding the effects of different study areas on CVI. Further comprehensive research is needed to describe the CVI characteristics of different regions of the choroid in CSC. Currently, there is no unified protocol for calculating CVI, and most studies use the macular region in the B-scan image for calculation. However, B-scan is a single cross-sectional image and cannot fully reflect the overall vascular structure changes of the choroid. Some researchers have used B-scan images to form three-dimensional reconstructions of choroidal vessels for CVI calculation, which represents the volumetric choroidal density and may more comprehensively reflect the ratio of CVI in a particular region compared to cross-sectional images [42]. In the sub-analysis based on the study area, two studies [37, 40] used the three-dimensional method, and WMD in this subgroup was lower than the macular subgroup (Fig 8E). Considering that both studies used a relatively large imaging range (6x6 mm and 24x20 mm), the reduction in WMD may be similar to our previous assumption, which is caused by the larger calculation area.

In the last decade, numerous studies have highlighted the critical role of the choroid in the development of CSC, although the exact pathology of the disease remains unclear. Spaide [13] et al. hypothesized that restrained outflow might be the reason for leaked fluids and suspected that congestion and anastomosis of the vortex vein are related to CSC. Other research has found that CVI is higher in regions with anastomosis [40]. CVI is influenced by the area of vascular diameter in OCT images, which could explain the increased CVI ratio observed in our meta-analysis. A dilated condition is thought to be associated with vascular permeability. When the choroidal vessel is hyperpermeable, increased tissue hydrostatic pressure is thought to compromise the normal function of the RPE and result in fluid leakage [43]. Furthermore, Liu [44]’s study revealed that polypoidal choroidal vasculopathy with choroidal hyperpermeability had a higher CVI than those without choroidal hyperpermeability, suggesting a potential correlation between CVI and the permeability state of the choroid. Therefore, to a certain extent, CVI may reflect the pathological changes of CSC eyes. Some studies have used CVI as an indicator to assess the treatment efficacy and choroid sensitivity to some suspected risk factors [45, 46].

CSC was considered unilateral, although some patients may experience SRD in both eyes. However, recent studies have shown that the healthy fellow eye of CSC may also undergo similar pathological changes. Thickened choroid has been observed in the fellow eye of CSC in a previous study [47]. Consistent with these findings, our meta-analysis also revealed a higher CVI in fellow eyes of acute CSC. An increased CVI ratio may imply that the fellow eye of CSC has undergone similar pathological changes, such as dilated vessels and vascular hyperpermeability. This finding supports the hypothesis that CSC may be a bilateral disease with symptoms in a single eye. Moreover, CVI changes in fellow eyes may be helpful to diagnose and classify the disease [48].

There is still some heterogeneity that we were unable to account for in this meta-analysis, which may be due to variations in method between the included studies. Different methodologies were used for calculating CVI, including the initial methods designed by Sonoda [18] and Agrawal [29], which use Image J software to process OCT images. This process involves binarizing the image and calculating the ratio of the luminal area to the choroidal area. Another recent method that has emerged uses artificial intelligence to automatically define and binarize the choroidal area, as proposed by Aslam [30] and Vupparaboina [49]. Although CVI is considered a reliable indicator because it is minimally affected by factors such as blood pressure and intraocular pressure [19], differences in methodology may lead to different results, as well as the use of different instruments in the studies, thus limiting a more comprehensive application in clinical practice. More research is needed to standardize the methods for calculating CVI and minimize methodological variations that may affect the results.

Several limitations of our study should be acknowledged. First, despite our efforts to include as many studies as possible, only 15 studies were included in this meta-analysis, most of which were conducted in Asia. This limited sample size and the predominantly Asian population may have affected the results of our analysis, and caution should be needed when generalizing our findings to other ethnicities or regions. It has been suggested that CSC may be more prevalent in Asian populations [50], which may further limit the applicability of our results. Secondly, there were variabilities between included studies in this meta-analysis due to the different OCT instruments and calculation protocols. Thirdly, although we excluded studies that included patients with a history of laser or ocular surgery, there were differences in the inclusion or exclusion criteria among the studies included. This suggests that some studies may have included patients who had undergone treatment, such as oral eplerenone. While the effects of some treatments on CVI need further research, this potential variability in patients may have potentially impacted the final results. Besides, other potential confounding factors were not considered, such as myopia’s effect on CVI [51].

In conclusion, our meta-analysis revealed that the CVI is higher in CSC eyes, suggesting that CVI could be a promising parameter for studying the vascular condition of the choroid. However, the application and development of CVI may be limited by differences in research protocols, such as area selection. Furthermore, more research is needed to define the characteristics of the choroid in CSC more precisely and to combine CVI with other imaging biomarkers to provide a more comprehensive description of the disease.

## Supporting information

S1 Table PRISMA checklist. (DOCX)

S1 File (DOCX)
