# Supplementary material for: Choroidal vascularity index in different types of central serous chorioretinopathy: A meta-analysis
Source: PLoS One. 2023 Jul 27;18(7):e0289186. doi: 10.1371/journal.pone.0289186 (PMC10374115; doi:10.1371/journal.pone.0289186)
Supplement: S1 File — (DOCX) [file pone.0289186.s002.docx]

Original articles available in the Pubmed can be accessed by the DOI numbers or URLs.

DOI numbers:

[10.1097/IAE.0000000000001040]

[10.1097/IAE.0000000000003256]

[10.1097/IAE.0000000000003452]

[10.1007/s00417-020-04740-6]

[10.1371/journal.pone.0257763]

[10.4103/JOCO.JOCO_225_20]

[10.1007/s00417-022-05588-8]

[10.1097/IAE.0000000000002585]

[10.1167/tvst.10.8.9]

[10.3390/diagnostics11050861]

[10.1167/iovs.62.15.19]

[10.3390/jcm12010150]

[10.1007/s00417-019-04524-7]

[10.3389/fmed.2022.967369]

URLs:

[https://pubmed.ncbi.nlm.nih.gov/27124882/]

[https://pubmed.ncbi.nlm.nih.gov/34267118/]

[https://pubmed.ncbi.nlm.nih.gov/35723918/]

[https://pubmed.ncbi.nlm.nih.gov/32415536/]

[https://pubmed.ncbi.nlm.nih.gov/34555122/]

[https://pubmed.ncbi.nlm.nih.gov/34084959/]

[https://pubmed.ncbi.nlm.nih.gov/35201403/]

[https://pubmed.ncbi.nlm.nih.gov/31259812/]

[https://pubmed.ncbi.nlm.nih.gov/34251422/]

[https://pubmed.ncbi.nlm.nih.gov/34064718/]

[https://pubmed.ncbi.nlm.nih.gov/34932061/]

[https://pubmed.ncbi.nlm.nih.gov/36614951/]

[https://pubmed.ncbi.nlm.nih.gov/31724090/]

[https://pubmed.ncbi.nlm.nih.gov/36160148/]

Study conducted by Wang can be found in URL

[http://www.tcsurg.org/article/10.3760/cma.j.issn.1005-1015.2019.04.008]
